# Pathogenic Intestinal Bacteria Enhance Prostate Cancer Development via Systemic Activation of Immune Cells in Mice

**DOI:** 10.1371/journal.pone.0073933

**Published:** 2013-08-26

**Authors:** Theofilos Poutahidis, Kelsey Cappelle, Tatiana Levkovich, Chung-Wei Lee, Michael Doulberis, Zhongming Ge, James G. Fox, Bruce H. Horwitz, Susan E. Erdman

**Affiliations:** 1 Division of Comparative Medicine, Massachusetts Institute of Technology, Cambridge, Massachusetts, United States of America; 2 Laboratory of Pathology, Faculty of Veterinary Medicine, Aristotle University of Thessaloniki, Thessaloniki, Greece; 3 Department of Immunopathology, Brigham and Women’s Hospital, Harvard Medical School, Boston, Massachusetts, United States of America; Northwestern University Feinberg School of Medicine, United States of America

## Abstract

A role for microbes has been suspected in prostate cancer but difficult to confirm in human patients. We show here that a gastrointestinal (GI) tract bacterial infection is sufficient to enhance prostate intraepithelial neoplasia (PIN) and microinvasive carcinoma in a mouse model. We found that animals with a genetic predilection for dysregulation of *wnt* signaling, *Apc* ^*Min/+*^ mutant mice, were significantly susceptible to prostate cancer in an inflammation-dependent manner following infection with *Helicobacter hepaticus*. Further, early neoplasia observed in infected *Apc* ^*Min/+*^ mice was transmissible to uninfected mice by intraperitoneal injection of mesenteric lymph node (MLN) cells alone from *H. hepaticus*-infected mutant mice. Transmissibility of neoplasia was preventable by prior neutralization of inflammation using anti-TNF-α antibody in infected MLN donor mice. Taken together, these data confirm that systemic inflammation triggered by GI tract bacteria plays a pivotal role in tumorigenesis of the prostate gland.

## Introduction

Only a small proportion of dysplastic and early neoplastic lesions arising in epithelia throughout the body actually advance into cancer [1–3]. Cancer happens when cells with malignant potential achieve all stimuli needed for their genetic and epigenetic evolution into malignancy [4]. It is now well documented that for many types of cancer a major contributor is cells and factors of the immune system [4–6]. In the prostate gland, several lines of evidence suggest that chronic inflammation drives the prostate cancer process [5,7,8], but its source remains elusive. Infectious agents, chemical or physical epithelial cell injury, hormonal imbalances and dietary factors have been proposed as the most probable causes of chronic inflammation in the prostate [8–10].

Determining whether prostate malignancy may be preventable by targeting underlying inflammation has particular importance since it is a leading cause of cancer-related disability and death among men. In the gastrointestinal (GI) tract, infections with pathogenic microbes, such as with *Helicobacter pylori*, lead to chronic inflammation (gastritis) and cancer (gastric adenocarcinoma) in humans and in mouse models [4]. Likewise, in immunocompromised mouse models the related lower bowel bacterium *Helicobacter hepaticus* (*H. hepaticus*) triggers inflammatory bowel disease (IBD) and colorectal carcinoma (CRC) [11–13]. In the prostate gland, however, few animal models exist to test possible direct or even indirect roles of pathogenic microbes in prostate malignancy [8,10,14].

In humans, the adenomatosis polyposis coli (Apc) gene is a critical tumor suppressor gene that prevents malignancy of prostate and other tissues [15]. Here, we use mutant mice that lack one copy of the *Apc* gene (*Apc* ^*Min/+*^ mice) and exhibit dysregulation of β-catenin and the wnt signaling pathway and are otherwise extensively used as a model of intestinal adenomas [16]. *Apc* ^*Min/+*^ mice spontaneously develop prostate intraepithelial neoplasia (PIN) and microinvasive carcinoma (microinvasive CA) with increasing age, mimicking the initial stages of prostate cancer seen in humans in their fifth or sixth decade of life [15,17]. We show that orogastric dosing with *H. hepaticus*, a non-colitigenic for this mouse model bacterial bowel colonizer [17], significantly increases prostate cancer. Importantly, development of prostate cancer was transmissible to uninfected animals solely by transfer of mesenteric lymph node cells from *H. hepaticus*-infected *Apc* ^*Min/+*^ donor mice.

## Results

### Orogastric infection with *Helicobacter hepaticus* increases risk of prostate carcinoma in male *Apc^Min/+^* mice

**Figure 1:**
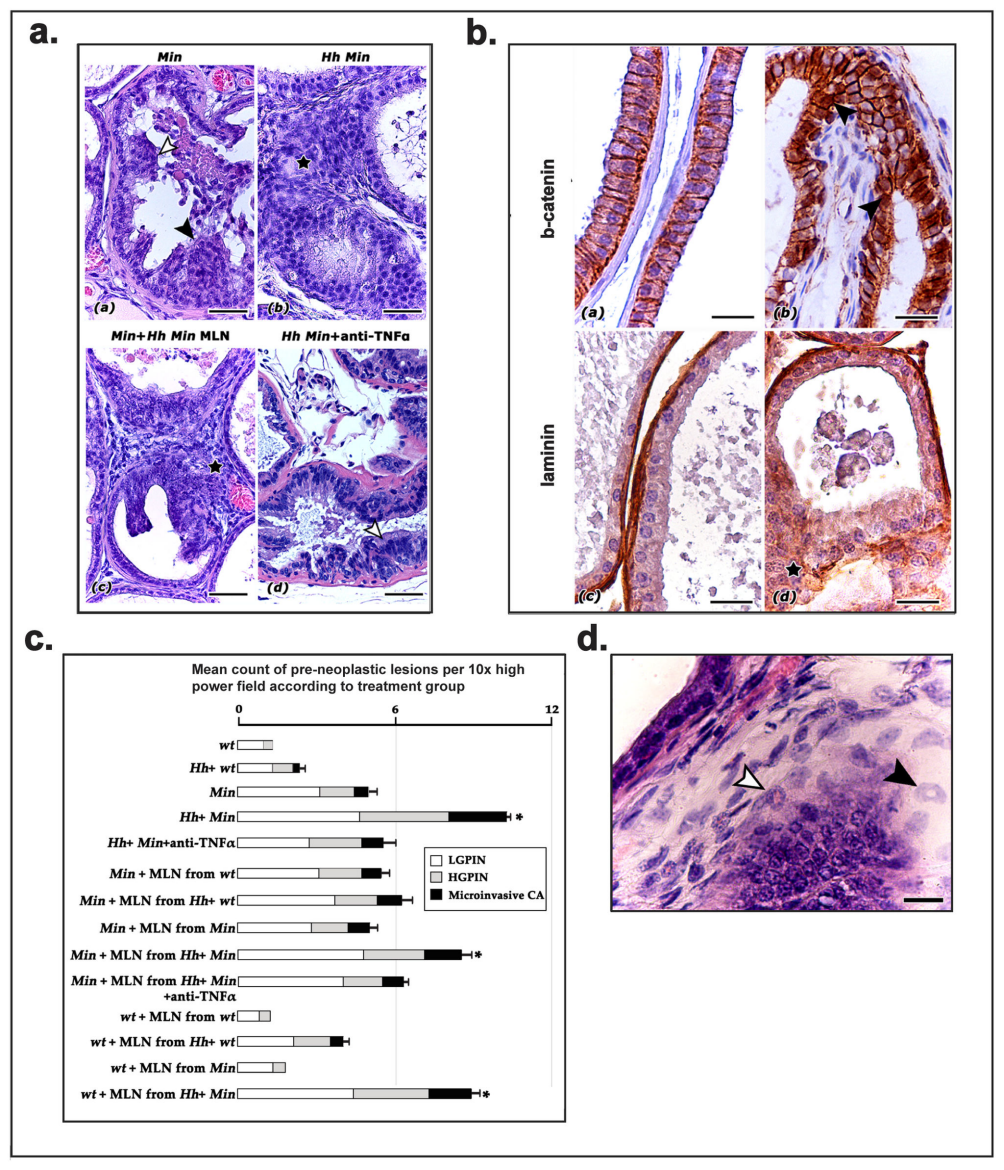
Clinically silent immune system alterations affect dorsolateral prostate (DLP) carcinogenesis in B6 *ApcMin* ^/+^ mice after GI infection with *H. hepaticus*. **A**) By the age of 12 weeks, *Apc* ^*Min/+*^ mice spontaneously develop focal low and high grade prostate intraepithelial neoplasia (LGPIN, white arrow-head; HGPIN, black arrow-head) but only rare microinvasive carcinoma (a). Aged-matched *Apc* ^*Min/+*^ mice with *H*. *hepaticus*, develop significantly more PIN and microinvasiveCA lesions (asterisk) characterized by highly atypical prostate gland epithelial cells which break through the basal lamina and infiltrate the adjacent stroma (b). When mesenteric lymph node cells from *H*. *hepaticus*-infected *Apc* ^*Min/+*^ mice are transferred into the peritoneal cavity of *H*. *hepaticus*-free *Apc* ^*Min/+*^ mice the recipient mice develop preneoplastic and early neoplastic lesions (microinvasiveCA-asterisk) comparable to those found in donor mice (c). Depleting TNF- α from *H*. *hepaticus*-infected *Apc* ^*Min/+*^ mice brings prostate neoplastic lesions (LGPIN, white arrow-head) occurrence to the level of *H*. *hepaticus*-free mice (d). Hematoxylin and Eosin. Bars=50 μm. **B**) Aberrant β-catenin and laminin immunostaining pattern in microinvasiveCA lesions in prostate cancer. DLP from *WT H*. *hepaticus-*positive mice (a and c), *H*. *hepaticus*-infected *Apc* ^*Min/+*^ mouse (b) and *H*. *hepaticus*-free *Apc* ^*Min/+*^ mouse transferred with mesenteric lymph node cells from *H*. *hepaticus*-infected *Apc* ^*Min/+*^ donor (d). Normal DLP glands had a normal β-catenin lateral epithelial cell membrane staining pattern (a) and an intact basal lamina (c). In contrast, microinvasiveCA lesions were characterized by cytoplasmic and nuclear (arrow-heads) stabilization of β-catenin (b) and absence of laminin (asterisk) suggestive of basal membrane degradation in malignant epithelial cell foci of incipient invasion. Hematoxylin counterstain, DAB chromogen. Bars=25 μm. **C**) Bar graph showing frequency of PIN and microinvasiveCA in treatment groups. The most significantly elevated prostate cancer occurrence is denoted by asterisk. Standard error bars correspond to microinvasiveCA statistical comparisons. **D**) DLP of *H*. *hepaticus*-free *Apc* ^*Min/+*^ mouse transferred with MLN cells from a *H*. *hepaticus*-infected *Apc* ^*Min/+*^ donor, HGPIN. Most myeloid precursor cells with ring-shaped nuclei residing in the stroma have the typical granulocytic lineage phenotype (black arrow-head) while fewer appear to be myelo/monocytic in type (white arrow-head). Bar=16 μm.

To determine the impact of intestinal infection, we used *Apc* ^*Min/+*^ mice that were cancer-free (young mice at age of 12 weeks had no prostate carcinoma or intestinal polyps) [17]. Mice were orally dosed with *H. hepaticus* or underwent sham dosing with media at six weeks of age. By 12 weeks of age, *Apc* ^*Min/+*^ mice infected with *H. hepaticus* displayed features of prostate carcinoma consistent with those observed in human males (Figure 1A, 1B; Table S1). *H. hepaticus*-infected *Apc* ^*Min/+*^ mice exhibited increased PIN (p<0.01) and microadenoCA (p<0.01) when compared with sham-dosed matched uninfected control *Apc* ^*Min/+*^ mice (Figure 1C and S1). Neither *H. hepaticus*-infected nor uninfected 12-week-old *wt* mice exhibited significant prostate pathology. Together these results demonstrate that infection of the GI tract with *H. hepaticus* rapidly enhances prostate carcinogenesis in genetically susceptible mice.

### Elevations of inflammatory cytokines occur without overt inflammatory disease in *H. hepaticus* –infected *Apc^Min/+^* mice

**Figure 2:**
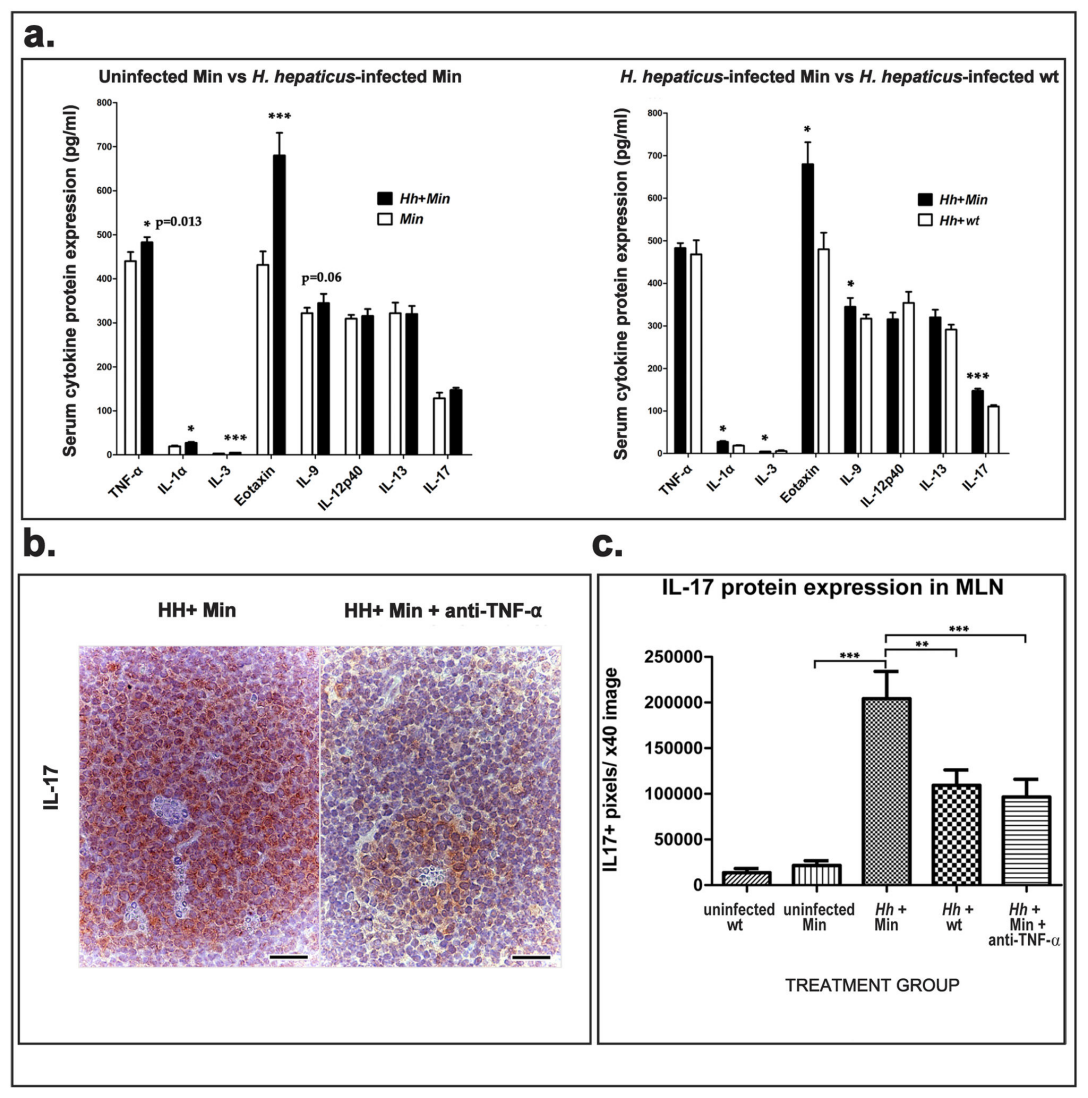
Intestinal *H. hepaticus* infection triggers systemic elevations in pro-inflammatory cytokines. **A**) Serum levels of pro-inflammatory cytokines were increased in *Apc* ^*Min/+*^ mice after *H*. *hepaticus* infection. Eotaxin, IL-3, TNF-α and IL-1α were significantly elevated in comparison with age-matched uninfected *Apc* ^*Min/+*^ controls. When compared with *H*. *hepaticus* infected *wt* mice, infected *Apc* ^*Min/+*^ mice had significantly higher IL-17, IL-1α, IL-3, eotaxin and IL-9 concentration in their blood serum. Bio-Plex Cytokine Assay used sera of *n*=5 mice per group. ***p<0.001; *p<0.05. **B**) Paracortical areas in mesenteric lymph nodes. *H*. *hepaticus* infected *Apc* ^*Min/+*^ mice at high risk of prostate cancer had large amounts of cytoplasmic and extracellular IL-17 that significantly decreased after depletion of TNF-α. IL-17-specific immunohistochemistry; Hematoxylin counterstain, DAB chromogen. Bars=25 μm. **C**) Morphometric assessment of IL-17 in immunohistochemically stained sections of mesenteric lymph nodes. Both *Apc* ^*Min/+*^ genetic status and intestinal infection by *H*. *hepaticus* significantly correlate with a TNF-α-mediated increase of IL-17 expression in the mesenteric lymph nodes. ***p<0.001; **p<0.01; *p<0.05.

We next tested whether *H. hepaticus* infection leads to increased inflammatory responses as previously shown [13]. We found that *H. hepaticus*-infected *Apc* ^*Min/+*^ mice exhibited increased systemic pro-inflammatory cytokines including tumor necrosis factor (TNF)-α (p=0.013), IL-1α (p=0.024), IL-3 (p=0.0009) and eotaxin (p=0.003) (Figure 2A left). Interestingly, prostates from *H hepaticus*-infected *Apc* ^*Min/+*^ mice lacked overt inflammatory disease but exhibited occasional mast cells, neutrophils, and also myeloid precursor cells bearing ring-shaped nuclei [18] (Figure 1D).

### Neutralization of inflammation inhibits *H. hepaticus* –induced prostate carcinoma in male *Apc^Min/+^* mice

Pro-inflammatory cytokine TNF-α was found to be elevated (p= 0.013) in sera after *H. hepaticus* infection for six weeks in three-month-old male *Apc* ^*Min/+*^ mice (Figure 2A). TNF-α is at the apex of a cascade of carcinogenic pro-inflammatory events [5]. To test whether inflammation is required for GI tract bacteria-triggered prostate pathology, we performed systemic neutralization of TNF-α using intraperitoneal injection of anti-TNF-α antibody for 3 weeks duration starting at 3 weeks post-infection (PI). We found that anti-TNF-α treatment led to significantly less (p<0.01) prostate pathology in *H. hepaticus*-infected *Apc* ^*Min/+*^ mice (Figure 1A, 1C and S1; Table S1). Taken together with earlier data, this indicated that *H. hepaticus*-triggered host inflammatory responses enhanced neoplastic effects in prostate tissue.

### Inflammatory cytokines were elevated in *H. hepaticus* -infected *Apc^Min/+^* mice but not in *H. hepaticus* -infected *wt* littermate mice

A feature of this mouse model is that *H. hepaticus*-infected *Apc* ^*Min/+*^ mice displayed significantly increased high grade PIN (HGPIN) and prostate carcinoma; however, matched *H. hepaticus*-infected *wt* littermate mice fail to develop significant PIN or carcinoma lesions (Figure 1B, 1C and S1, Table S1). Upon analysis of serum cytokine levels, we discovered significant differences in protein levels of IL-1α (p=0.02) and IL-17 (p=0.0005) between *Apc* ^*Min/+*^ and the *wt* littermate mice (Figure 2A right). Likewise, *in situ* labeling of IL-17 showed increased IL-17+ expression levels in MLN in *H. hepaticus*-infected *Apc* ^*Min/+*^ mice but not in the *H. hepaticus*-infected *wt* controls (Figure 2B and 2C). Treatment of *H. hepaticus*-infected *Apc* ^*Min/+*^ mice with anti-TNF-a antibody significantly decreased mRNA gene expression (p<0.01) and protein (p<0.001) levels of IL-17A in MLN (Figure 2B and 2C). Taken together, these accumulated data raised the possibility that systemic differences originating from bacteria-triggered inflammation of GI tract origin may contribute to carcinogenic processes in distant tissues such as prostate.

### Prostate cancer risk is transmissible to uninfected recipients using mesenteric lymph node (MLN) cells from *H. hepaticus* -infected *Apc^Min/+^* mice

**Figure 3:**
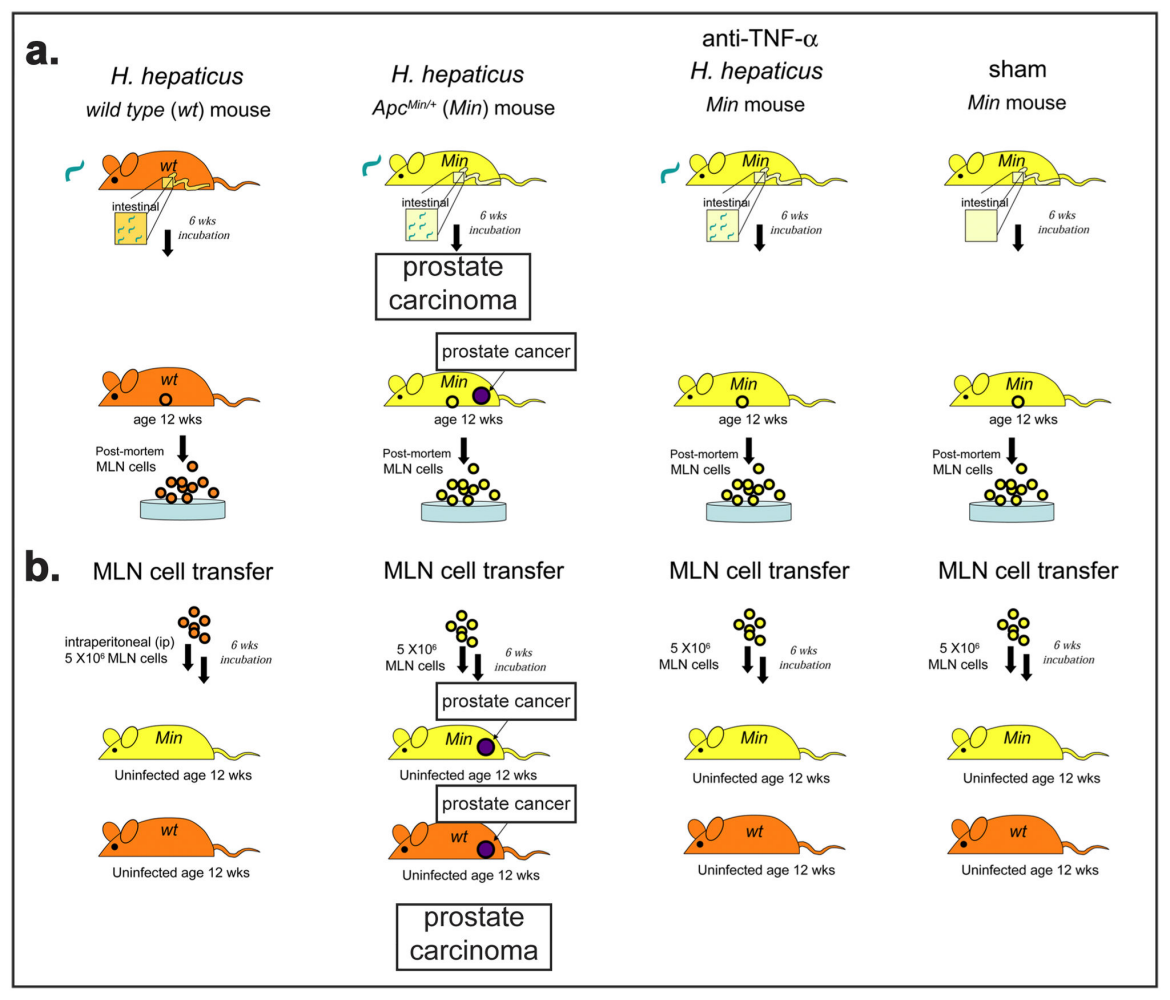
Transfer of mesenteric lymph node (MLN) cells from *H hepaticus*-infected mice is sufficient to recapitulate prostate cancer in uninfected mice. Schematic illustration of MLN cells transfer experiments designed to test the hypothesis that local GI tract immune network events cause systematic immune imbalances that determine the fate of preneoplastic lesions in topographically distant epithelia such as those of the prostate gland. Trials used 6 mice per group. Experiments were done in duplicate.

Knowing that GI tract origin inflammatory response is necessary for prostate cancer in our model, we hypothesized that cells within gut-associated lymph nodes (MLN) may be pivotal in prostate cancer in this model. In order to test this hypothesis, we utilized cell transfer of MLN collected from *H. hepaticus*-infected or sham-dosed donor *Apc* ^*Min/+*^ or *wt* littermate mice (Figure 3). For this experiment, all recipient animals were *Helicobacter* *sp* -free male *Apc* ^*Min/+*^ or *wt* littermates. Male mice of age 6-8 weeks underwent ip injection of 5X10^6^ single cell suspension in HBSS per mouse of cells gathered from mesenteric lymph nodes of *H. hepaticus*-infected or sham-dosed *Apc* ^*Min/+*^ or *wt* mice.

Consequently, we found significantly increased low (p<0.05) and high grade (p<0.01) PIN in uninfected *Apc* ^*Min/+*^ mice that had merely received MLN cells from *H. hepaticus*-infected *Apc* ^*Min/+*^ mice (Figure 1A, B, C and S1) at 6 weeks after ip injection, when compared with MLN donor cells from sham-dosed *Apc* ^*Min/+*^ mice. These uninfected *Apc* ^*Min/+*^ that received cells from *H. hepaticus*-infected *Apc* ^*Min/+*^ displayed features of preneoplasia and carcinoma consistent with those observed in *H. hepaticus*-infected *Apc* ^*Min/+*^ mice (Figure 1A), and resembling those seen in human males. In addition, uninfected *wt* recipient mice that received lymph node cells from *H. hepaticus*-infected *Apc* ^*Min/+*^ donor mice also had increased LGPIN (p<0.001), HGPIN (p<0.001) and adenocarcinoma (p<0.001) (Figure 1C and S1; Table S1) compared to uninfected wt mice that received lymph node cells from *H. hepaticus*-free *Apc* ^*Min/+*^ donor mice showing that genetic predisposition of prostate epithelia was not specifically required for carcinogenic effect of *H. hepaticus* infection. This transmissibility feature showed that *H. hepaticus*-activated cells were sufficient for neoplastic processes in extra-intestinal glands such as prostate.

### Neutralization of inflammation prevents MLN cell transmissibility of prostate cancer risk to uninfected mice

Finally, we tested whether neutralizing inflammation was sufficient to inhibit MLN-cell mediated transmissibility of prostate cancer in this mouse model. We utilized cell transfer of MLN collected from *H. hepaticus*-infected *Apc* ^*Min/+*^ mice that had been treated with anti-TNF-a antibody for 3 weeks (Figure 3). For this experiment, all recipient animals were *Helicobacter* *sp* -free male *Apc* ^*Min/+*^ or *wt* littermate animals. Male mice of age 6-8 weeks underwent ip injection of 5X10^6^ single cell suspension in HBSS per mouse of cells gathered from mesenteric lymph nodes of *H. hepaticus*-infected *Apc* ^*Min/+*^ mice. As predicted, we found no significant differences between uninfected *Apc* ^*Min/+*^ mice vs uninfected *Apc* ^*Min/+*^ mice that had received MLN from anti-TNF-α-treated mice (p>0.05). Likewise, there was significantly less LGPIN (p<0.01) and HGPIN (p<0.05) in uninfected *Apc* ^*Min/+*^ recipients of MLN from anti-TNF-treated *H. hepaticus*-infected mice when compared with *Apc* ^*Min/+*^ recipient mice of MLN from *H. hepaticus*-infected *Apc* ^*Min/+*^ mice (Figure 1C and S1; Table S1). These data indicate that the pro-inflammatory role of the MLN cells is the underlying cause of the observed prostate cancer risk transmissibility.

## Discussion

In the present study, we test young male *Apc* ^*Min/+*^ mice on a C57BL/6J background as an experimental model to probe associations of inflammation with carcinogenesis in the prostate. As with humans where prostate cancer risk has a strong genetic component, *Apc* ^*Min/+*^ mutant mice are genetically predisposed to epithelial neoplasia [13,16,17]. We and others have shown in the past that *Apc* ^*Min/+*^ mice also progressively develop prostate cancer on the basis of increased PIN lesions matching that seen in human males [15,17]. In our studies, *Apc* ^*Min/+*^ mice develop early neoplastic lesions in the dorsolateral prostate as early as at 3 months of age preceding evident intestinal polyps. At age = 6 months, 40% of *Apc* ^*Min/+*^ mice develop microinvasive adenocarcinoma while 10-20% also have more advanced prostate cancer lesions [15]. This aspect gives the *Apc* ^*Min/+*^ mouse utility as a model to examine progression of pre-neoplastic and early neoplastic lesions in the human prostate [1–3].

An important finding of this study is that infection with *H. hepaticus*, a bacterium that colonizes primarily the large intestine of C57BL/6 *ApcMin* ^*/+*^mice without overt typhlocolitis [13,19–21], significantly increased the incidence of PIN and microinvasive adenocarcinoma lesions in the prostate. As expected, 3-months-old *Apc* ^*Min/+*^ mice infected with *H. hepaticus* had neither overt IBD nor large adenomatous polyps yet formed in their bowels (data not shown). However, even at this young age, these mice did exhibit increased systemic pro-inflammatory cytokines, such as TNF-α, IL-1α, IL-3, eotaxin and IL-9, compared to their uninfected control counterparts, indicative of immune system activation. These data taken together with previous evidence that *H. hepaticus* infection is able to modulate tumorigenesis at extra-intestinal sites [21,22], suggest that clinically silent GI tract immune network events triggered by gut microbiota may cause systematic immune imbalances. In turn, these imbalances may impact the fate of preneoplastic lesions in topographically distant epithelia such as those of the prostate gland.

The interrelated roles of the elevated proinflammatory cytokine milieu and the inflammatory cellular component found in the prostate cancer prone *H. hepaticus*-infected *Apc* ^*Min/+*^ mice are interesting. Within the prostate gland, PIN and microinvasive adenocarcinoma featured mast cells, neutrophils, and myeloid precursor cells bearing ring-shaped nuclei. TNF-α was previously proven essential in mast cell recruitment and tumorigenesis in *Apc* ^*Min/+*^ mice [13,17,23]. The release of factors including TNF-α and various proteases from mast cells residing in the prostate stroma may have an important role in tumorigenesis [10].

TNF-α and IL-1α have been together implicated in a NF-κB-mediated signal cascade that inhibited apoptosis and increased the proliferation of epithelial cells in human prostate preneoplasia and cancer [24]. In addition, IL-1α promotes the abnormal proliferation of human prostate epithelial cells through insulin growth factor- and fibroblast growth factor-7-related growth signals [25,26]. Eotaxin and IL-3 regulate bone marrow maturation, release and tissue trafficking of myeloid precursor cells [27] such as those seen in prostate glands of *H. hepaticus*-infected *Apc* ^*Min/+*^ mice. Potential roles of eotaxin, IL-3 and associated myeloid precursor cells in epithelial carcinogenesis warrant further investigation. The powerful pro-inflammatory role of IL-9 is increasingly appreciated. Th9 cells constitute the latest addition expanding the T-helper subtype array. IL-9 stimulates the growth of hematopoietic cells, especially mast cells and the secretion of several chemokines that recruit additional cells to inflamed tissues [28]. The important role of IL-9 in autoimmunity and allergy [28] may provide important clues, especially in the light of recent hypotheses that connect prostate inflammation with cancer. Prostate epithelial cell injury may then cause loss of tolerance to normal prostatic antigens and consequently lead to a self-perpetuating autoimmune reaction [10]. In the present study, our finding that neutralization of TNF-α was sufficient to counteract prostate cancer risk enhancement in a mouse model suggests that this cytokine has a central role in creating a pro-tumorigenic systematic inflammatory macro-environment that allows the progression of cancer in the prostate gland.

Another pro-inflammatory cytokine we found significantly elevated by comparing *H. hepaticus*-infected *Apc* ^*Min/+*^ mice with *H. hepaticus*-infected *wt* littermate mice was Il-17A. The significant differences in Il-17 were of particular interest because we have previously shown IL-17+ cells in mesenteric lymph node (MLN) of mice after infection with *H. hepaticus* [20]. Likewise, IL-17 has been implicated in infection-triggered bowel cancer development in *Apc* ^*Min/+*^ mice [29,30]. The correlation of the *Apc* ^*Min/+*^ mouse genotype with systematic and MLN elevations in Il-17 expression may have a genetic basis, since Il-17-associated deficits in the immune cell function of this mouse model have been reported [30]. Importantly, recent evidence from both preclinical mouse [31] and clinical human studies support a significant role for Il-17 in prostate carcinogenesis [32,33].

To provide experimental proof for this hypothesis and retain the complex immunological interplay of the various cell populations we chose to transfer total MLN cell populations into mice. Immunological activities within systemic lymph node tissue [20] may be the link connecting GI microbial flora with universally pro-tumorigenic systematic alterations of the immune system. Surprisingly, we found that increased prostate cancer risk is transmissible to uninfected *Apc* ^*Min/+*^ recipients of MLN cells only taken from *H. hepaticus*-infected *Apc* ^*Min/+*^ donors. There was no evidence of *H. hepaticus* organisms in prostate tissue of transplant recipient mice using PCR or by *in situ* examination using silver stain (data not shown), but translocation of *H. hepaticus* organisms or their antigens from intestines to lymph nodes remain a possible contributing factor. Our finding that MLN cells taken from anti-TNFα-treated *H. hepaticus*-infected *Apc* ^*Min/+*^ lost the prostate cancer promoting effects provides evidence that inflammation enhanced this carcinogenic process.

In conclusion, a possible role for microbes in prostate malignancy has been suspected [9] but remained unproven until now. Here we have demonstrated that ingestion of *H. hepaticus* alone enhanced prostate carcinogenesis in *Apc* ^*Min/+*^ mice. Our finding that inflammation enhanced this cancer forming process adds mechanistic support to the well-documented benefits of non-steroidal anti-inflammatory drugs (NSAIDs) in human patients. Remarkably, uninfected mice that received only lymph node cells became highly susceptible to prostate cancer. This observation that prostate cancer is inducible by distilled cells from another organ may help explain why etiopathogenesis of prostate cancer in humans has been difficult to elucidate. It remains to be definitely determined whether other GI tract microbes in humans, such as *H pylori*, may have a similar effects in prostate and other non-gastrointestinal cancers of humans.

## Materials and Methods

### Experimental animals

All animals were housed and handled in Association for Assessment and Accreditation of Laboratory Animal Care (AAALAC)-accredited facilities with diets, experimental methods, and housing as specifically approved by the Institutional Animal Care and Use Committee. The MIT CAC (IACUC) specifically approved the studies as well as the housing and handling of these animals. *Apc* ^*Min/+*^ mice on a C57BL/6J background were originally obtained from the Jackson labs and bred in-house to provide *Apc* ^*Min/+*^ mice and *wildtype* (*wt*) littermates for experimental recipients and donors. *Helicobacter* -free status of the mice was confirmed by PCR using *Helicobacter* genus-specific primers as previously described [20].

### Experimental *Helicobacter hepaticus* infection

A total of 44 experimental mice were dosed at 6 weeks of age with *H. hepaticus* (strain 3B1, ATCC #51449) and then housed separately in a bio-containment area within the same animal facility. *H. hepaticus* was grown under microaerobic conditions, prepared, and confirmed pure as described elsewhere [11,20]. Experimental mice received 0.2ml of fresh inoculum by gastric gavage every other day for a total of three doses. Cecum, mesenteric lymph node (MLN), and prostate were collected 6 weeks post-infection at necropsy and analyzed by PCR to confirm (bowel) or exclude (MLN, prostate) colonization.

### Experimental design

A total of 96 *Apc* ^*Min/+*^ mice and 60 wt mice were included in various treatment regimens or as experimental controls. Experiments were conducted using two separate trials with 5-6 mice each, as specified in Table S1. Sixty *Apc* ^*Min/+*^ mice and 40 wt mice age of 6 weeks were injected intraperitoneally with 5 x 10^6^ MLN cells suspended in HBSS. The cell donor mice were male *H. hepaticus*-infected six weeks earlier, or *Helicobacter* -free C57BL/6 *wt* or *Apc* ^*Min/+*^ mice.

### TNF-α neutralization

A total of 24 *H. hepaticus* infected *Apc* ^*Min/+*^ or *wt* mice at age of 9 weeks (starting at 3 weeks after *H. hepaticus* infection) received anti-TNF-α antibody (clone XT-3; BioExpress, Lebanon, NH) at 200µg per mouse three times weekly for three weeks as described previously [13].

### Detection of systemic cytokine protein expression

Serum cytokine levels of 5-6 animals in each experimental group were analyzed using the Bioplex Pro Mouse Cytokine assay system (BioRad, Hercules, CA) according to the manufacturers protocol. Briefly, serum samples were diluted using the sample diluent kit, incubated with antibody coated beads followed by a secondary antibody incubation and analyzed in duplicates on a Bio-Plex 200 system (BioRad, Hercules, CA).

### Histologic evaluation and Immunohistochemistry

The formalin-fixed tissues were processed and paraffin embedded 5µm tissue-sections were stained by H&E, Warthin-Starry silver stain or IHC and evaluated for prostate lesions by a veterinary pathologist blinded to sample identity. The preneoplastic and early neoplastic prostate lesions, detected based on recent consensus report criteria [34], were quantitatively assessed as described previously by Poutahidis et al (2009) [17].

Rabbit primary antibodies used for immunohistochemistry included anti-β-catenin (Thermo, Fisher Scientific/Lab Vision, Fremont, CA), anti-laminin (Thermo, Fisher Scientific/Lab Vision, Fremont, CA) and anti-IL17 (Santa Cruz Biotechnology, Santa Cruz, CA). Antigens were retrieved with Protease 1 (Roche Diagnostics/Ventana Medical Systems, Tucson, AZ) for laminin detection. Heat-induced antigen retrieval was performed with citrate buffer pH6 for β-catenin or with EDTA buffer pH8 for IL17 detection. Primary antibody binding was detected with goat anti-rabbit Zytochem Plus HRP Polymer (Zytomed Systems, Berlin, Germany). Signal was detected with diaminobenzidine (Invitrogen, Camarillo, CA) and tissues were counterstained with hematoxylin.

For the quantification of IL-17 expression in MLN, thirty 40x high power field images of IHC-stained sections were captured per treatment group. Ten images from each group were randomly selected for counts. Automatic calculation of the numbers of pixels corresponding to IL-17 in images appeared in a histogram after processing each image with the threshold color plug-in of the ImageJ image processing and analysis program (NIH, Bethesda, MD). Morphometric counts were recorded as the number of IHC labeled pixels per image.

### Statistical analyses

The prevalence of LGPIN, HGPIN and micro-adenoCa between groups was compared by the Kruskal-Wallis one-way ANOVA and Dunn’s post-test. Direct comparisons were made by the Mann–Whitney U test. Serum cytokine data analysis was performed using the 2-tailed student’s t-test. IL-17 immunohistochemically-positive image pixel count data were evaluated for normality with Shapiro-Wilk test and analyzed with one way analysis of variance (ANOVA) and Tukey’s multiple comparison post test. Statistical analyses were performed with Graphpad Prism version 5.0 for Windows, GraphPad software, San Diego, CA, USA; a p-value < 0.05 was considered statistically significant.

## Supporting Information

Figure S1 **Statistical analyses of prostate pathology in treatment groups.** Treatment groups match those portrayed in Figure 1. ***p<0.001; **p<0.01; *p<0.05.
(TIF)

Table S1 **Frequency of prostate pathology types within treatment groups.**
(DOC)
