# Supplementary figures and images for: Pathogenic Intestinal Bacteria Enhance Prostate Cancer Development via Systemic Activation of Immune Cells in Mice
Source: PLoS One. 2013 Aug 26;8(8):e73933. doi: 10.1371/journal.pone.0073933 (PMC3753256; doi:10.1371/journal.pone.0073933)

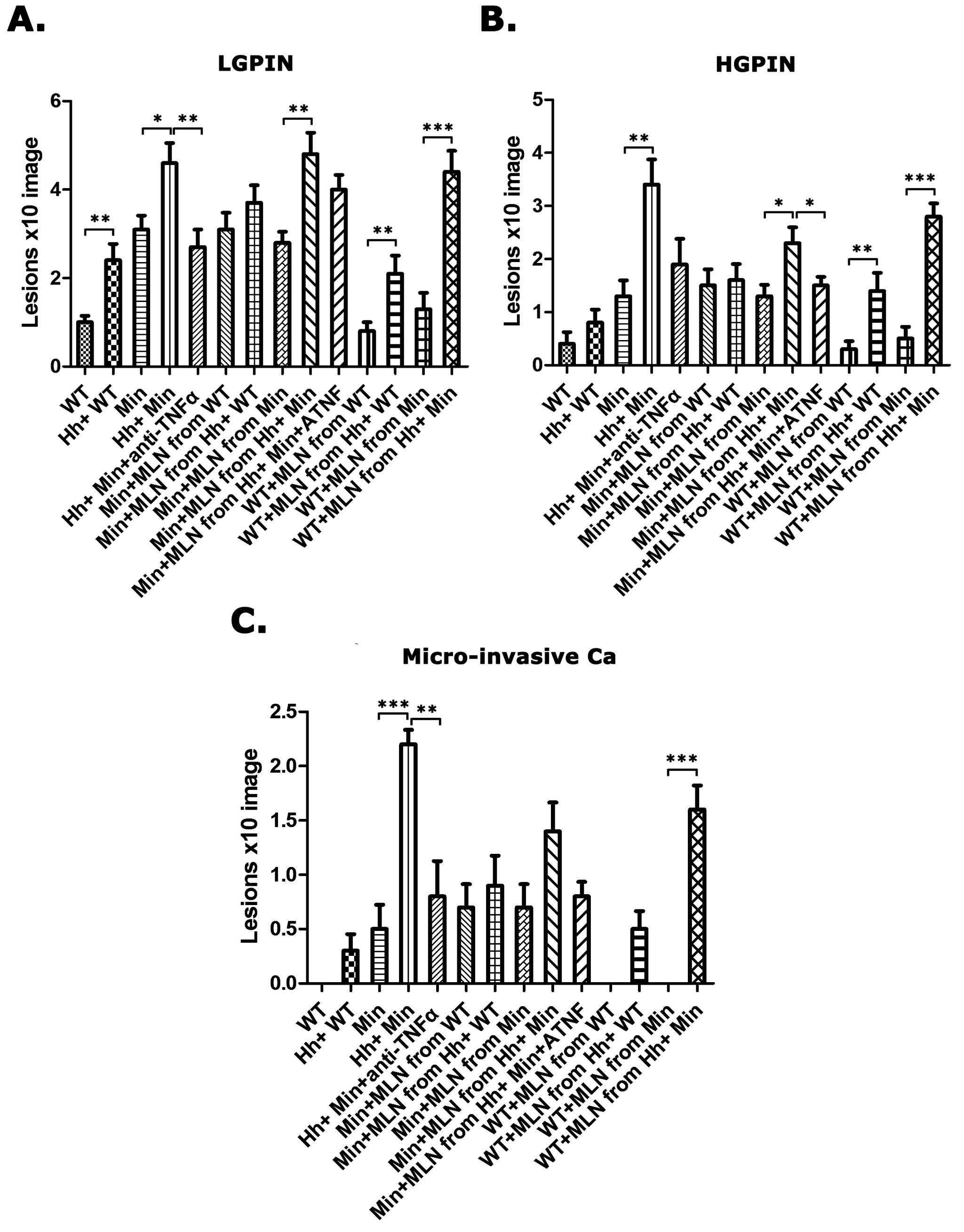

Supplement: Figure S1 — Statistical analyses of prostate pathology in treatment groups. Treatment groups match those portrayed in Figure 1. ***p<0.001; **p<0.01; *p<0.05. (TIF) [file pone.0073933.s001.tif]
