# Supplementary material for: Pathogenic Intestinal Bacteria Enhance Prostate Cancer Development via Systemic Activation of Immune Cells in Mice
Source: PLoS One. 2013 Aug 26;8(8):e73933. doi: 10.1371/journal.pone.0073933 (PMC3753256; doi:10.1371/journal.pone.0073933)
Supplement: Table S1 — Frequency of prostate pathology types within treatment groups. (DOC) [file pone.0073933.s002.doc]

**Table S1. Frequency of prostate pathology types within treatment groups.**

| Experimental Group |  | Type of Lesion | | |
| --- | --- | --- | --- | --- |
|  |  | LGPIN | HGPIN | Microinvasive CA |
| WT |  | 30% (3/10) | 10% (1/10) | 0% (0/10) |
| Hh+ WT |  | 70% (7/10) | 30% (3/10) | 20% (2/10) |
| Min |  | 100% (12/12) | 66% (8/12) | 33% (4/12) |
| Hh+ Min |  | 100% (12/12) | 100% (12/12) | 100% (12/12) |
| Hh+ Min+anti-TNFα |  | 100% (12/12) | 58% (7/12) | 41% (5/12) |
| Min+MLN from WT |  | 100% (12/12) | 58% (7/12) | 41% (5/12) |
| Min+MLN from Hh+ WT |  | 100% (12/12) | 91% (11/12) | 58% (7/12) |
| Min+MLN from Min |  | 100% (12/12) | 75% (9/12) | 50% (6/12) |
| Min+MLN from Hh+ Min |  | 100% (12/12) | 100% (12/12) | 91% (11/12) |
| Min+MLN from Hh+ Min+anti-TNFα |  | 100% (12/12) | 83% (10/12) | 58% (7/12) |
| WT+MLN from WT |  | 30% (3/10) | 20% (2/10) | 0% (0/12) |
| WT+MLN from Hh+ WT |  | 100% (10/10) | 50% (5/10) | 30% (3/10) |
| WT+MLN from Min |  | 50% (5/10) | 30% (3/10) | 0% (0/10) |
| WT+MLN from Hh+ Min |  | 100% (12/12) | 100% (12/12) | 100% (12/12) |
